# Special Issue “A Commemorative Issue in Honor of József Szejtli: Advances in Cyclodextrin Chemistry and Its Applications”

**DOI:** 10.3390/ijms26189246

**Published:** 2025-09-22

**Authors:** Lajos Szente, Éva Fenyvesi

**Affiliations:** CycloLab, Illatos ut 7, 1097 Budapest, Hungary; lajos.szente@cyclolab.hu

This Special Issue is dedicated to the memory of the late Dr. József Szejtli, on the 90th anniversary of his birth. It is intended, in a small way, to recognize Dr. Szejtli’s outstanding contributions to the scientific research and technical developments that have resulted from the industrial implementation of new technologies associated with the molecular-level encapsulation of substances with cyclodextrins (CDs).

Dr. Szejtli, along with his early co-workers, pioneered techniques leading to the industrial application of CDs. Without Szejtli’s pioneering work, the CD industry would likely not be as advanced as it is today. 

Szejtli graduated from the Technical University of Budapest in 1956, focusing on the field of polysaccharide chemistry. Having received his Ph.D. in starch chemistry, Szejtli decided to continue his career abroad. He received a fellowship in Norway at the Royal Seaweed Research Institute in 1963. Soon after this, he spent two years in Potsdam, East Germany, where he worked on the formation and structural aspects of iodine/amylose inclusion complexes: a step closer to the development of “CD complexes”, the topic of his life’s work. From the cold and foggy East German climate, Szejtli moved to a much more sunny and warm location, Havana in Cuba, where he worked for 4 years as a science advisor to the Cuban Government and professor of biochemistry at the University of Havana.

Returning to Hungary, Szejtli successfully convinced the management of Chinoin Pharmaceuticals to organize his Cyclodextrin Laboratory in 1972, and to support his vision to explore the application potentials of CD technology. Szejtli frequently alluded to the importance of sunshine in polysaccharide chemistry, saying that “as long as the sun shines, green plants perform photosynthesis, [and] there is enough inexpensive raw material, i.e., starch, available for CD technology”.

His first task was to refute the toxicity of cyclodextrins, which was a false conclusion reached in an animal experiment performed previously using incompletely purified cyclodextrins. He was the first to prepare 14C-labeled cyclodextrin and proved that beta-cyclodextrin is not absorbed by the gastrointestinal tract. With these two important findings, he opened up the path for real progress.

He also recognized that the main hurdle to application was the high price due to the lack of industrial production. With the help of a fermentation research group, he scaled up the laboratory method to a hundred-ton scale in the case of beta-cyclodextrin and to the scale of tens of tons for alpha- and gamma-cyclodextrin.

Having obtained sufficient cheap cyclodextrins, Szejtli’s CD research group tirelessly investigated and explored every possible field of CD applications, including in the pharmaceutical, food, cosmetics, analytical, and agrochemical fields. Between 1975 and 1980, Szejtli not only headed and supervised the research at his lab, but also traveled extensively to convince colleagues abroad—from university scientists to chief executives of industrial organizations—that CDs held great commercial promise. Szejtli truly believed that the sharing and consolidation of the information on CDs in the technical literature was an efficient way to move from research toward more commercial applications. 

Between 1982 and 1988, he wrote two seminal monographs dealing with all possible aspects of CD technology: “Cyclodextrins and their Inclusion complexes” and “Cyclodextrin Technology”. 

Dr. Szejtli has received much recognition for his work, including an Academy Award from the Hungarian Academy of Sciences, the internationally awarded Moet Hennessy Science of Art prize, and the Széchenyi prize—the highest recognition a Hungarian citizen can achieve. Szejtli succeeded at the greatest challenge a scientist faces: he created commercial products from his ideas. Hence, József Szejtli can undoubtedly be considered the Godfather of Cyclodextrins, a real pioneer of CD science and technology in many ways, such as the following:

- He initiated the first systematic, comprehensive research and development programs on CDs.
- He organized the first international CD symposium (in Budapest, 1981).
- He wrote the first seminal monograph on CDs.
- He established the first *CD Newspaper*, a monthly newsletter on CDs.
- He established the first private independent research and development firm, CycloLab, dedicated solely to cyclodextrin applications.

Szejtli’s contribution to CD technology is recognizable even today. He published over 500 technical papers, was the inventor of over 100 patents, and authored four books and several book chapters.

József Szejtli, a scientist, pioneer, entrepreneur, mentor, and motivator in CD science and technology, richly deserves the recognition of his peers. We, the Guest Editors, hope that this recognition and acknowledgment of Dr. Szejtli’s scientific merit is well expressed by the content of this Special Issue.

